# Fatal Infantile Hepatic Dysfunction Associated With TRMU Gene Mutation and Aggravated by Cytomegalovirus Infection: A Unique Case

**DOI:** 10.7759/cureus.104646

**Published:** 2026-03-04

**Authors:** Saydé Khattar, Pamela Rizk, Faissal Tleiss

**Affiliations:** 1 Pediatrics, Faculty of Medical Sciences, Lebanese University, Beirut, LBN; 2 Pediatrics, Faculty of Medicine and Medical Sciences, University of Balamand, Beirut, LBN; 3 Neonatology, Nini Hospital, Tripoli, LBN

**Keywords:** cytomegalovirus coinfection, infantile hepatopathy, liver failure, mitochondrial disease, trmu mutation

## Abstract

Transient infantile liver failure due to tRNA 5-methylaminomethyl-2-thiouridylate methyltransferase (TRMU) gene mutation is a rare mitochondrial disease (MD) that typically presents within the first few months of life. We present the case of a 50-day-old female infant who was admitted with jaundice, hepatomegaly, lactic acidosis, and signs of liver dysfunction. Extensive metabolic and infectious investigations revealed a homozygous TRMU gene mutation and a high cytomegalovirus (CMV) viral load. The patient was treated with intravenous ganciclovir, supportive liver management, and metabolic correction; however, her clinical course was complicated by hepatic failure, coagulopathy, anemia, and ultimately cardiac arrest. This case represents the first reported instance of fatal infantile liver failure associated with a TRMU mutation, with CMV infection as a possible aggravating factor, from Lebanon.

## Introduction

Mitochondrial diseases (MDs) represent a heterogeneous group of inherited disorders characterized by dysfunction of the mitochondrial respiratory chain, impairing cellular energy production. These conditions can manifest at any age and affect virtually any organ system, often presenting as multisystem disorders [1]. Mutations can arise in either mitochondrial DNA (mtDNA) or nuclear DNA (nDNA), both of which encode components essential for oxidative phosphorylation [1].

Among the nuclear-encoded genes involved in mitochondrial function, the tRNA 5-methylaminomethyl-2-thiouridylate methyltransferase (TRMU) gene plays a crucial role. It encodes a mitochondrial enzyme that modifies specific mitochondrial transfer RNAs (mt-tRNAs), thereby supporting accurate mitochondrial protein translation [2,3]. Mutations in the TRMU gene have been linked to transient infantile liver failure, a rare MD with variable clinical presentations [2].

Epidemiological estimates suggest that MDs collectively affect approximately 1 in 5,000 individuals. The clinical expression of these disorders can vary even among individuals carrying the same mutation, with manifestations ranging from isolated symptoms in adulthood to severe, life-threatening, multi-organ involvement in neonates [4]. In neonates, liver involvement is a common presentation, frequently manifesting as cholestasis, coagulopathy, hepatosplenomegaly, or even fulminant hepatic failure [5].

Several nuclear gene mutations affecting mitochondrial protein translation have been associated with neonatal liver dysfunction, including GFM1, TUFM, and TRMU mutations [6]. Complicating factors, such as cytomegalovirus (CMV) infection, can further aggravate MDs. CMV is the most frequent congenital viral infection and may present with a range of symptoms, including hepatitis, growth restriction, jaundice, hepatosplenomegaly, sensorineural hearing loss, and neurological abnormalities [7]. In affected neonates, early diagnosis via polymerase chain reaction (PCR) and antiviral treatment with ganciclovir or valganciclovir can improve outcomes [8].

In this report, we present the first known case from Lebanon of a fatal infantile liver failure associated with a homozygous TRMU mutation, further complicated by concurrent CMV infection. We describe the patient’s clinical course, diagnostic workup, and outcome, and we discuss how this genetic-infectious interplay contributed to disease progression.

## Case presentation

A 50-day-old female infant, weighing 3.35 kg, born at 36 weeks of gestation via cesarean section due to intrauterine growth restriction (IUGR), presented to the emergency department with tachypnea and one episode of non-bloody, non-bilious, non-projectile vomiting. The infant was afebrile, passing urine and stools normally. Antenatal history was unremarkable, and maternal screening was negative for HIV, hepatitis B virus, and group B Streptococcus. Birth weight was 2.7 kg, with Apgar scores of 9 and 10 at 1 and 5 minutes, respectively.

Upon arrival, the patient was pale, hypotonic, and tachypneic. Fresh blood was noted in the stool. She was admitted to the neonatal intensive care unit for further evaluation. Her initial vital signs are summarized in Table 1.

**Table 1:** Vital signs on presentation. The patient presented with fever, tachypnea, and severe hypoglycemia, while other vital parameters remained within age-appropriate ranges, indicating acute metabolic instability requiring urgent evaluation.

Parameter	Value	Reference range (neonates)
Temperature	38.3°C	36.5-37.5°C
Heart rate	150 bpm	100-160 bpm
Respiratory rate	80 bpm	30-60 bpm
Blood pressure	80/55 mmHg	65-85/45-55 mmHg
Oxygen saturation (SpO₂)	97%	>94%
Capillary blood glucose (HGT)	20 mg/dL	40-60 mg/dL (hypoglycemia)

**Figure 1:**
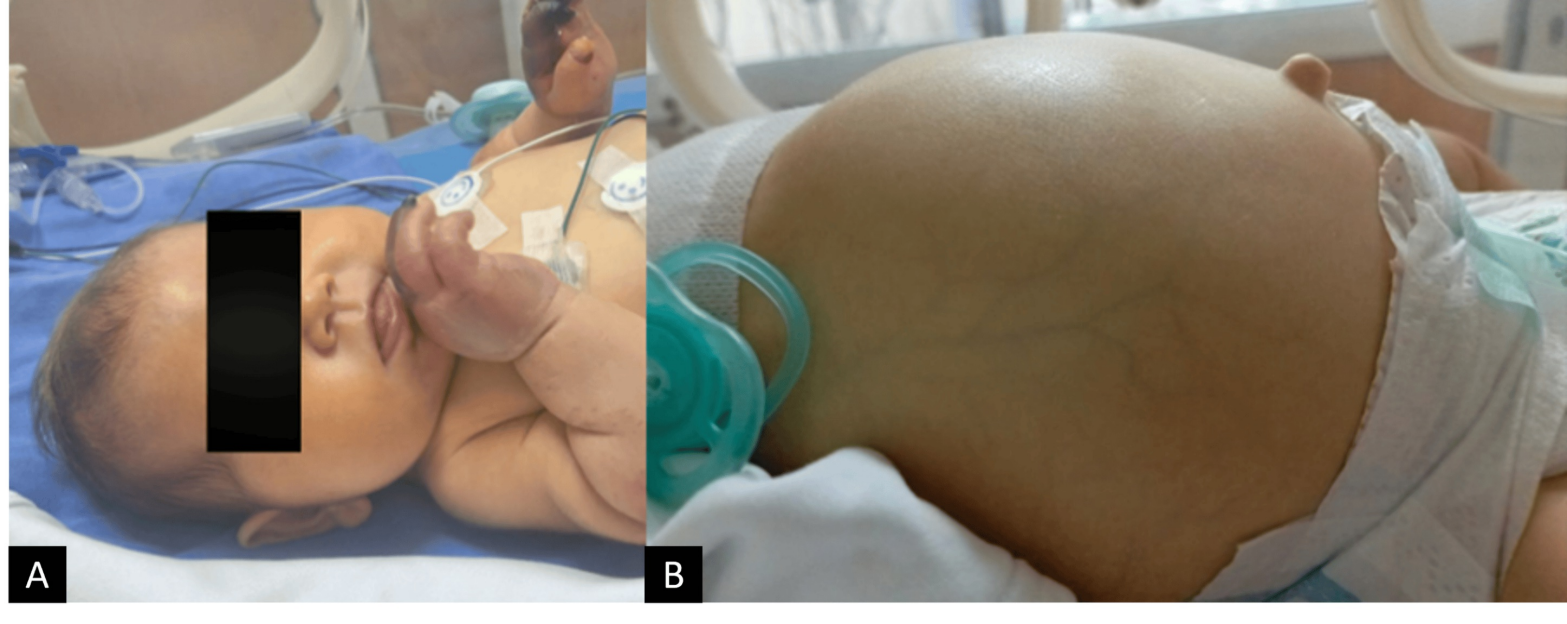
(A) Jaundice and (B) hepatomegaly development on day 2 of presentation.

**Figure 2:**
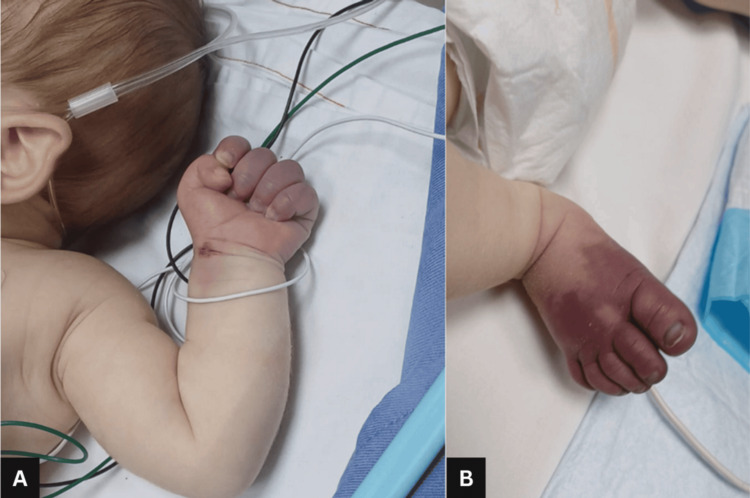
Cyanosis over the bilateral (A) upper and (B) lower extremities on day 1 of presentation.

**Figure 3:**
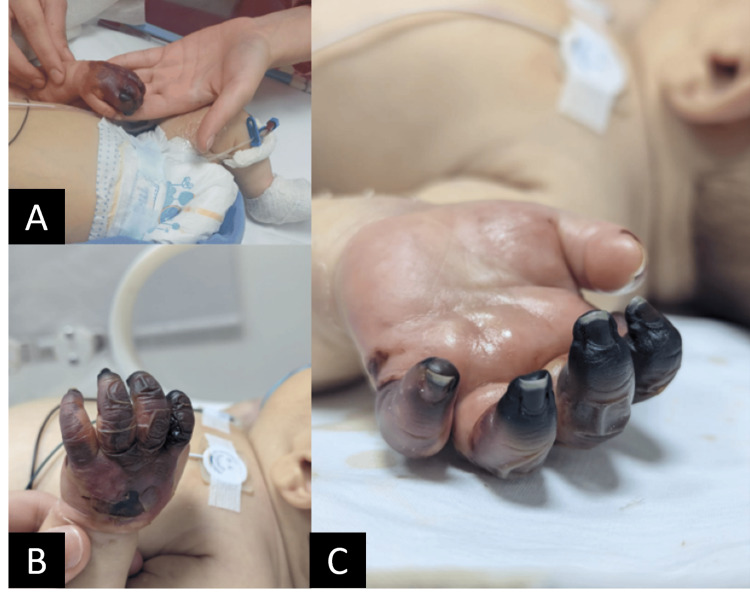
Progression of peripheral upper limb discoloration from (A) cyanosis to (B) ischemia to (C) necrosis.

**Figure 4:**
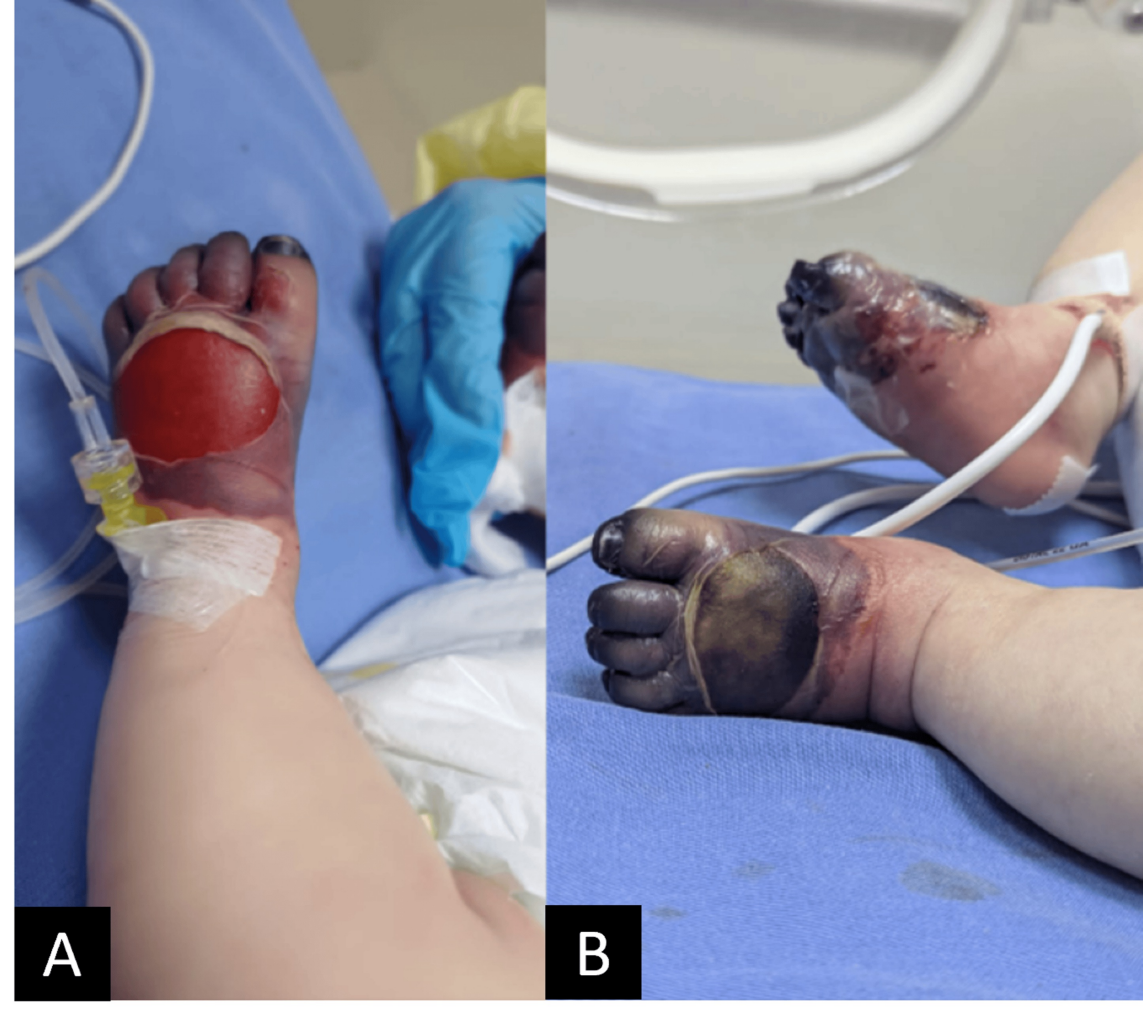
Progression of peripheral lower limb discoloration from (A) cyanosis into (B) necrosis.

Initial management included a 2 mL/kg intravenous bolus of 10% dextrose water and paracetamol. On physical examination, hepatosplenomegaly was initially absent. However, by day 2, the infant developed visible jaundice and hepatomegaly (Figure 1). Cyanosis over the bilateral upper and lower extremities developed on day 1 and progressed to necrosis during hospitalization (Figures 2-4).

A full hematologic and biochemical workup was performed on admission (see Table 2).

**Table 2:** Full laboratory investigations on admission. Laboratory findings demonstrate metabolic acidosis with marked lactic elevation, anemia, inflammatory activation, and severe hepatic dysfunction with coagulopathy, hyperbilirubinemia, hyperammonemia, and hypoalbuminemia, indicating acute multisystem involvement.

Test	Result	Reference range (neonates)	Interpretation
Complete blood count (CBC)			
White blood cell (WBC)	12,800/µL	5,000-12,000/µL	Slightly elevated
Hemoglobin	8.9 g/dL	11-14 g/dL	Anemia
Hematocrit	27.40%	33%-43%	Low
Mean corpuscular volume (MCV)	96.5 fL	80-100 fL	Normal
Platelets	376,000/µL	150,000-450,000/µL	Normal
Neutrophils	42%	30%-60%	-
Lymphocytes	44%	20%-40%	Slightly elevated
Inflammatory markers			
C-reactive protein (CRP)	12 mg/dL	<5 mg/dL	Elevated
Procalcitonin	0.77 ng/mL	<0.5 ng/mL	Elevated
Arterial blood gas (ABG)			
pH	7.22	7.35-7.45	Acidemia
pCO₂	12.7 mmHg	35-45 mmHg	Respiratory compensation
HCO₃⁻	5.2 mmol/L	22-28 mmol/L	Low
Lactic acid	19.6 mmol/L	0.5-2.2 mmol/L	Markedly elevated
Liver function tests			
Total bilirubin	12 mg/dL	0.2-1.2 mg/dL	Elevated
Direct bilirubin	8.5 mg/dL	<0.3 mg/dL	Elevated
International normalized ratio (INR)	6	0.8-1.2	Coagulopathy
Aspartate aminotransferase (AST) (serum glutamic-oxaloacetic transaminase, SGOT)	372 IU/L	10-40 IU/L	Elevated
Alanine aminotransferase (ALT) (serum glutamic pyruvic transaminase, SGPT)	350 IU/L	7-56 IU/L	Elevated
Gamma-glutamyl transferase (GGT)	99 IU/L	12-43 IU/L	Elevated
Ammonia	143 µmol/L	11-35 µmol/L	Hyperammonemia
Protein profile			
Total proteins	28.9 g/L	60-80 g/L	Low
Albumin	20 g/L	35-50 g/L	Hypoalbuminemia

Further investigations included an infectious workup, which showed negative bacterial cultures (blood, urine, and stool), but blood PCR was positive for CMV, with a viral load of 25,000 copies/mL. Intravenous ganciclovir was initiated, and the patient completed 29 days of treatment until repeat PCR testing was negative.

Doppler abdominal ultrasound showed progressive hepatosplenomegaly, with liver span reaching 7.1 cm and spleen 6.3 cm. Brain imaging (ultrasound) was unremarkable, showing no calcifications or malformations.

A comprehensive coagulation and hematology panel was also performed (Table 3).

**Table 3:** Hematologic and coagulation workup. The coagulation profile shows elevated D-dimer, hypofibrinogenemia, reduced natural anticoagulants, and multiple clotting factor deficiencies, consistent with severe coagulopathy and impaired hepatic synthetic function.

Parameter	Result	Reference range	Interpretation
D-dimer	0.9 mcg/mL	<0.5 mcg/mL	Elevated
Fibrinogen	0.58 g/L	1.5-4.0 g/L	Low
Haptoglobin	<0.08 g/L	0.3-2.0 g/L	Low
Protein C	12.50%	55%-160%	Low
Protein S	53%	60%-150%	Near low
Factor II	Low	50%-150%	Decreased
Factor V	Low	50%-150%	Decreased
Factor VII	Low	50%-150%	Decreased
Factor X	Low	50%-150%	Decreased
Antiphospholipid antibodies	Negative	Negative	-
Anticardiolipin antibodies	Negative	Negative	-

Metabolic and endocrine screening were mostly normal (Table 4), with a few exceptions.

**Table 4:** Metabolic and endocrine workup. The metabolic evaluation reveals markedly elevated ferritin and alpha-fetoprotein levels with abnormal amino acid findings, while thyroid and most metabolic parameters remain within normal limits, suggesting metabolic and hepatic stress without primary endocrine dysfunction.

Test	Result	Reference range	Interpretation
Uric acid	1.5 mg/dL	2.0-5.5 mg/dL	Low-normal
Triglycerides	33 mg/dL	30-150 mg/dL	Normal
Homocysteine	13.5 µmol/L	<15 µmol/L	Normal
Alpha-1 antitrypsin	0.89 g/L	0.9-2.0 g/L	Borderline
Thyroid-stimulating hormone (TSH)	5.2 µIU/mL	0.5-6.0 µIU/mL	Normal
Free T4	1.05 ng/dL	0.9-2.3 ng/dL	Normal
Acylcarnitine profile	Elevated alanine and phenylalanine	-	Abnormal amino acids
Ferritin	2,961 ng/mL	25-200 ng/mL	Markedly elevated
Alpha-fetoprotein	6,412 ng/mL	<500 ng/mL	Markedly elevated

Follow-up laboratory testing was performed throughout the admission, reflecting an unstable clinical course of the patient (Table 5).

**Table 5:** Follow-up laboratory testing throughout the admission. Serial laboratory monitoring demonstrated fluctuating cytopenias, persistent hepatic dysfunction, intermittent coagulopathy, and episodic metabolic derangements, reflecting an unstable clinical course with partial biochemical improvement followed by recurrent deterioration. WBC: white blood cell, Hb: hemoglobin, MCV: mean corpuscular volume, CRP: C-reactive protein, BUN: blood urea nitrogen, SGOT: serum glutamic-oxaloacetic transaminase, SGPT: serum glutamic pyruvic transaminase, GGT: gamma-glutamyl transferase.

	Day 2	Day 4	Day 6	Day 7	Day 9	Day 12	Day 15	Day 18	Day 21	Day 24	Day 27	Day 30	Day 35	Day 37	Day 40	Day 44
WBC (/uL)	8.8	6.4	13	16	10	11	11	16	14	14	16	19	7.3	8	7.6	16.4
Hb (g/dL)	7.7	8	7.2	5.6	10	10	9.4	9.9	8.8	8.2	7.6	11	8.6	9.9	5.4	8.36
MCV (fL)	91	89	90	91	85	86	87	88	91	87	85	85	86	88	90	84
Platelets (/uL)	,	-	137	169	167	218	264	333	346	374	393	339	188	97	9.9	57
Neutrophils (%)	58	40	41	20	32	31	32	38	43	42	39	41	30	14	51	40
CRP (mg/dL)	22	25	18	16	11	7.8	7	6.8	5.6	6	4	4	5.7	3.8	5.3	10.7
BUN (mg/dL)	5.7	3	3	3	4	3	3.8	3.1	3.7	3.2	3	3.2	3.3	4.7	4	7
Creatinine (mg/dL)	0.3	0.23	0.2	0.3	0.25	0.15	0.15	0.17	0.16	0.18	0.3	0.2	0.2	0.16	0.29	1.07
SGOT/SGPT/GGT (IU/L)	110/194	85/196	75/164/57	70/163/63	73/141/ 58	116/152/49	160/187/51	188/230/56	182/220/53	157/225/67	203/247/74	215/257/85	372/35/99	292/241/83	-	335/181/51
Protein (g/L)/albumin(g/L)	34/24	41/29	39/30	45/33	45/31	48/34	52/37	56/39	53/37	60/41	62/40	62/41	68/43	56/33	-	49/32
PT/PTT	-	-	73/96	57/80	41/65	48/66	41/63	56/79	50/80	50/64	56/71	49/54	72/82	90/140	90/140	-
Bilirubin T/D (mg/dL)	-	-	6.8/3.9	6.7/4	7/4.2	45842	7.8/4.6	45874	7.7/4.8	7.4/4.7	7.9/4.8	9.7/6.3	8-Dec	12/8.5	-	-
Lactic acid (mmol/L)	19.6	-	-	14	-	-	-	-	-	13.8	9.4	-	-	-	-	-
Ammonia (umol/L)	-	-	-	-	-	80	-	143	-	80	-	94	-	140	-	-

Due to the severity and unclear etiology of her presentation, whole-exome sequencing (WES) was performed and revealed a homozygous pathogenic mutation in the TRMU gene (NM_018006.4:c.262G>A). This mutation is associated with mitochondrial dysfunction, early-onset liver failure, and neurological deterioration.

During hospitalization, the patient developed worsening liver failure, anemia, thrombocytopenia, and coagulopathy, requiring transfusions of packed red blood cells, platelets, and fresh frozen plasma. She later became hypotensive and anuric. Dopamine and furosemide were initiated. On the 35th day of admission, the patient developed hematemesis followed by cardiac arrest, and despite resuscitative efforts, she was pronounced deceased.

Verbal informed consent was obtained from the patient's legal representative for the publication of this case report and any accompanying clinical details. Written consent was not required in accordance with institutional practice for case reports, and verbal consent was considered appropriate as the patient’s legal representative was not physically available to provide written documentation at the time of manuscript preparation. A final copy of the manuscript was sent to the patient’s legal representative prior to any submission process.

## Discussion

This case describes a 50-day-old female infant who presented with hepatomegaly, jaundice, lactic acidosis, coagulopathy, and hypoalbuminemia, all suggestive of hepatic failure. Further evaluation revealed a pathogenic homozygous TRMU mutation, which, along with elevated CMV viral load, contributed to progressive clinical deterioration despite supportive care and antiviral therapy.

In the neonatal period, other metabolic conditions such as galactosemia and tyrosinemia type 1 may present with similar hepatic findings. However, TRMU-related liver failure is distinct in its association with MD and often follows a transient course. While previous literature has described reversible liver dysfunction in cases of TRMU mutations, with approximately 28 such cases reported globally [9], our case unfortunately had a fatal outcome, suggesting a more severe phenotype or modifying factors. This may represent the first fatal case associated with a homozygous TRMU variant reported in Lebanon.

In earlier studies, patients typically carried compound heterozygous mutations in the TRMU gene [2], while our patient was found to have a homozygous missense mutation. Elevated serum lactate levels supported the presence of mitochondrial respiratory chain dysfunction. Several cases in the literature report that, with early recognition and supportive treatment, surviving infants can experience full recovery and reach normal developmental milestones [10,11]. However, our patient’s course was complicated by CMV infection, which may have acted synergistically to worsen the mitochondrial dysfunction and accelerate hepatic failure.

While many infants with TRMU-related disease present between two and four months of age, the clinical spectrum can range from mild cholestasis to fulminant hepatic failure with multi-organ involvement [12]. In contrast to our case, one previously reported infant with compound heterozygous mutations in TRMU exhibited only transient cholestasis and recovered completely [13]. This illustrates the significant variability in disease expression, possibly influenced by genetic, infectious, or environmental modifiers.

The fatal course in our case, marked by worsening hepatic failure, coagulopathy, anemia, necrosis of extremities, and eventual cardiac arrest, highlights the importance of early genetic screening in neonates presenting with unexplained liver dysfunction. The coexistence of a mitochondrial gene defect and a congenital viral infection likely amplified the severity of the disease and may explain the deviation from the more typical transient course seen in other TRMU-related cases.

## Conclusions

A wide range of heritable disorders, collectively referred to as MD, invariably affect how the respiratory chain in the mitochondria functions and how energy is produced inside cells. A clinically oriented genetic approach is advised in order to arrive at a diagnosis quickly and accurately. The genetic study of the case in question revealed a compound homozygous mutation in the TRMU gene associated with liver failure, particularly in the first few months of life.

As demonstrated in our case, where the patient presented with complicated liver failure, anemia, and thrombocytopenia, followed by hematemesis, cardiac arrest, and regrettably refusing resuscitation, mutations in these genes may be linked to a much milder presentation, but in most cases, this condition is temporary, with a complete clinical and biochemical resolution within the next months or years of life. However, it was fatal for our patient.
